# Cancer Care of Children, Adolescents and Adults With Autism Spectrum Disorders: Key Information and Strategies for Oncology Teams

**DOI:** 10.3389/fonc.2020.595734

**Published:** 2021-01-19

**Authors:** Delphine Vuattoux, Sara Colomer-Lahiguera, Pierre-Alain Fernandez, Marine Jequier Gygax, Marie-Louise Choucair, Maja Beck-Popovic, Manuel Diezi, Sabine Manificat, Sofiya Latifyan, Anne-Sylvie Ramelet, Manuela Eicher, Nadia Chabane, Raffaele Renella

**Affiliations:** ^1^ Division of Autism Spectrum Disorders and Related Conditions, Department of Psychiatry, Lausanne University Hospital, Lausanne, Switzerland; ^2^ Institute of Higher Education and Research in Healthcare, University of Lausanne, Lausanne, Switzerland; ^3^ Department of Oncology, Lausanne University Hospital, Lausanne, Switzerland; ^4^ Pediatric Hematology-Oncology Unit, Division of Pediatrics, Department “Woman-Mother-Child”, Lausanne University Hospital, Lausanne, Switzerland; ^5^ Division of Medical Oncology, Department of Oncology, Lausanne University Hospital, Lausanne, Switzerland; ^6^ Department “Woman-Mother-Child”, Lausanne University Hospital, Lausanne, Switzerland

**Keywords:** outcome disparities, autism (autism spectrum disorders), cancer, neurodevelopmental disorders, supportive care, oncology (general), medical oncology, pediatric oncology

## Abstract

Delivering optimal cancer care to children, adolescents and adults with ASD has recently become a healthcare priority and represents a major challenge for all providers involved. In this review, and after consideration of the available evidence, we concisely deliver key information on this heterogenous group of neurodevelopmental disorders, as well as recommendations and concrete tools for the enhanced oncological care of this vulnerable population of patients.

## Introduction

Providing care to children, adolescents and adults with cancer requires communicating effectively in an age- and development-specific way. With the increase in the complexity of healthcare systems and sophistication of available cancer therapies, individuals with neurodevelopmental challenges, including autism spectrum disorders (ASD), constitute a particularly vulnerable group for disparities in access and outcomes. Oncology providers need to become progressively more educated to the challenges posed by caring for the ASD population and have the necessary tools to implement effective communication and favorable environments for screening, diagnosis and therapy of cancer in this setting.

Therefore, our multidisciplinary expert panel of oncologists, nurses, neurologists and autism mental health specialists (cf. **Supplementary Material 1**) reviewed the available evidence and provide a) a concise synthesis of the evidence relevant for the optimal oncological care of patients with ASD and b) a discussion of recommendations and strategies and on how teams can enhance their care and provide support for these patients and their families/caregivers in their cancer trajectory.

## Definition, Prevalence, and Causes of ASD: Required Basic Knowledge for All Oncology Teams

ASD is a neurodevelopmental condition characterized by social communication deficits, restricted, repetitive and stereotypical patterns of behavior, interests, or activities, and sensory atypicalities (1). It is associated with various levels of intellectual and motor functioning and verbal skills, anxiety, and often with symptoms of other neurodevelopmental disorders, such as Attention Deficit Hyperactivity Disorder (ADHD) (2, 3). A subgroup of patients with ASD without intellectual disability (ID) were referred to/diagnosed as having Asperger’s syndrome prior to the revision leading to the DSM-5 classification. In this population, where a diagnosis is often made later in life, autism without ID remains unrecognized because clinical manifestations can a) overlap with other comorbidities of ASD or other psychiatric disorders, and b) patients, in majority females, can display sophisticated autism compensatory strategies (e.g., social camouflaging) (4, 5).

ASD is a most likely complex polygenic condition, with both *de novo* and rare inherited variants acting on a background of common genetic polymorphisms. Twin and family studies support the existence of heritable risk factors in ASD (6–8). Associations have been found in genes involved in early brain development (incl. synapse formation/stabilization and neurotransmission), in particular in the gamma-aminobutyric acid (GABA)-ergic system (9). Numerous pre-/perinatal risk factors and environmental exposures have also been suggested (10–13). Two studies also showed an increased prevalence of cancer among children with ASD, and recent genome/exome-wide sequencing studies of *de novo* and recurrent copy number variations in ASD and cancer have suggested an overlap in genes conferring risk for autism and cancer (14–16).

On an epidemiological level, prevalence of ASD has been steadily increasing over the last two decades, and it is now estimated at 16·8/1,000 (i.e., 1/59) in children of 8 years of age in the United States, with a shift in gender ratios (M:F) recently dropping from 8:1 to of 3:1 (17–19). The increase in prevalence has been attributed, but not exclusively, to a growth in awareness of the disorder with younger ages at diagnosis (20, 21). Additionally, a 2013 revision of the diagnostic criteria part of the “Diagnostic and Statistical Manual of Mental Disorders” (DSM-5) classification has led to an increase of diagnoses of individuals with less severe symptoms (1, 22). Thus, in the past decade, ASD prevalence increased almost 3.5 fold among children aged 2–17 years, mainly accounted for by an eightfold increase of ASD without intellectual disability (17). Alternatively, the increase prevalence has been speculated to be linked to a combination of genetic and environmental risk factors (23, 24). ASD sex ratio variations have been associated with the use of more accurate diagnostic criteria or could be related to sexually dimorphic neuroimmune system activation and the microbiome (25, 26).

## Healthcare and Cancer Care for Individuals With ASD

Within the population of individuals with high mental health needs, those with ASD represent a heterogeneous and challenging group in virtually every healthcare area (4, 27). During their lifetime, 70% of patients with ASD will be diagnosed with a medical or psychiatric comorbidity, with a negative impact on educational/employment outcomes (28, 29). In comparison to the general population, individuals with ASD have higher prevalence rates for almost all medical and psychiatric conditions, especially dyslipidemia, obesity, hypertension, gastrointestinal disorders, autoimmune conditions, asthma, allergies, infections, epilepsy, sleep disorders, depression, and visual and hearing impairments (30–35). How these comorbidities influence the risk of developing cancer, how they impact its early detection and the delivery of oncological care, remains unknown.

A recent study based on the United States National Survey of Children’s Health identified an approximately four times higher odds of unmet health care needs in children with ASD when compared to children without disabilities, disproportionately to children with other disabilities (36). Compiling data from research across the last decade, two recent reviews underline the limited availability of specific healthcare services for families with a child with ASD, and the lack of ASD-specific training in healthcare providers (37, 38).

As a possible consequence of underutilization of healthcare resources leading to unmet needs, an increased overall mortality has been observed in the ASD population (39–41). This is associated with complications related to different comorbidities including suicide, accidental death resulting from unsafe behaviors, use of supplemental medications such as atypical antipsychotics, poor nutrition, and insufficient/inappropriate use of healthcare resources (42–45). Specifically, the odds ratio of death from a neoplasm was estimated to be close to double in individuals with ASD (1.83 in females, 1.79 in males) when compared than non-ASD controls (41). This latter analysis was a matched case cohort study linking two nationwide population-based Swedish registers including 27’122 individuals with ASD (n=6240 low-functioning, n=20882 high functioning) and almost 3 million controls. Interestingly, individuals with low-functioning ASD had a higher OR of death from a neoplasm (2.12, 1.25–3.61 95% CI) than their high-functioning counterparts (1.75, 1.39–2.21 95% CI). As the incidence of cancer in ASD has been measured to be globally equal to that of the general population, differences in outcomes are most likely attributable to disparities in optimal cancer care, ranging from limited access to screening, delayed diagnosis and ineffective therapy.

While an early and correct diagnosis of ASD facilitates the deployment of adequate conditions for cancer care, this is possibly not sufficient. Oncology healthcare providers might lack a sufficient understanding of these neuro-developmental conditions, and/or be unaccustomed to the challenges caused by admitting an individual with ASD to their services (i.e., difficulties with emotional rigidity, inflexibility, misunderstanding/misinterpreting social situations, among others). Education and training in communication have been identified as a priority to improve cancer care for all individuals with limited intellectual abilities (46). This is particularly relevant for patients with ASD who generally are heavily dependent on their proxy-network (e.g., parents, siblings, family members in extenso, and other support providers) for their daily activities and health maintenance. National programs to improve education and communication skills of providers have been initiated in several countries, including the United Kingdom’s National Health Service’s “Right to be heard” campaign (47).

## Delivering Cancer Care to Individuals With ASD: Potential Obstacles and Solutions

Oncology teams expect concrete strategies to improve their understanding of the challenges faced by in/out-patients with ASD, and to identify their individual needs and the necessary environmental arrangements. Raising awareness and providing specific education for all members of the interprofessional team is the required first step. The abovementioned recent United Kingdom national policy addresses the rationale for a mandatory nation-wide staff training in identifying and specifically supporting patients with autism/learning disabilities and their associated conditions (47). Among the innovative proposals is the concept that, because face-to-face educational opportunities trump e-learning, individuals with ASD should contribute to this training, by advocating for the community’s needs and challenges.

While we await that other implement similarly ambitious national strategies, it is key for oncologists and their teams to have access to information on “red flags”. We therefore summarized the main features hinting to ASD in **Table 1**, categorizing them by age group.

**Table 1:** *Signs and symptoms of ASD at different ages; screening and diagnostic tools* (adapted from (48, 49)).

Age (years)	Social communication	Restriction and or repetitive behavior and interest	Screening*	Diagnosis**
Toddlers 0–3	- Delay/absence in language prerequisites (e.g., social smile, eye contact, response to his/her name, joint attention, babbling, pointing, social anticipation and social gestures) - Delay/atypical language development (no words at 16 months, no spontaneous association of two-words at 24 months) - Loss/regression of language or social skills - Lack of pretend or imaginative play	- Stereotypies (e.g., hand flapping)/body mannerisms (e.g., toe walking) - Atypical play (e.g., lining up or flipping object or toys) - Frequent atypical behavior with reactive tantrum after minimal stimulation/constraint - Attachment to unusual objects (e.g., stones, boxes, cigarette butts) - Atypical sensory exploration (e.g., putting objects close to the eyes, sniffing them, putting them in the mouth) - Excessive reactions or indifference to physical proximity or contact, to pain or temperature	Toddler: *M-CHAT CSBS* Child:*SRS SCQ*	*ADOS-2 Toddler ADI-R* (> 4 yo)
School age children 4–12	- Deficit in socio-emotional reciprocity (e.g., lack of theory of mind, reduced sharing of emotions or affects) - Difficulties with pragmatic language (e.g., initiate and maintain a normal conversation that is far removed from one’s interests, limited or, conversely, very elaborate conversation and vocabulary about one’s interests, failure in back-and-forth conversation, difficulties with sarcasm and metaphors, stereotyped and repetitive use of language, strange prosody) - Social difficulties and peculiarities with other children (e.g., difficulty playing with other children, developing and maintain friendships, adjusting behavior to different social contexts) - Difficulties in relationships with adults (e.g., excessive intensity/distance)	- Difficulties in managing change, unpredictable situations, or transitions between activities - Narrow or circumscribed interests (e.g., dinosaurs, numbers) - Special preference for atypical interests or activities (e.g., collecting, calculating, making lists) - Atypical sensory reactions (e.g., inappropriate or adverse responses to specific sounds or textures) - Excessive reactions or indifference to pain or temperature - Motor particularities (e.g., odd gestures, stereotypies, difficulties in motor coordination)	*SCQAQ child CAST*	*ADOS-2 (1-2-3) ADI-R*
Teenagers13–18	- Social awkwardness and difficulties in social relationships/behavior (e.g., hardship in making and maintaining friendships, facilitated relationships with adults or younger people, lack of autonomy, “social naivety”) - Continued difficulties with social communication (e.g., inappropriate calmness, limited or, conversely, very elaborate conversation and vocabulary about their one’s interests) - Inappropriate social communication (e.g., too much familiarity, “professorial” explanations) - Difficulties to understand other’s point of view		*AQ/EQSCQ*	*ADOS-2 (3)ADI-R*
Adults	- Continued social awkwardness, difficulties with employment/emotional life - Possible “social camouflaging” especially for women (e.g., efforts to hide autistic features in social situations)	- Obsessive-compulsive behavior (OCD) - Cognitive rigidity - Narrow interests	*AQ, EQ RAADS SCQ*	*ADOS-2 (4)ADI-R*
**Factors masking ASD at any age** - Co-morbid psychiatric conditions (e.g., anxiety, hyperactivity, mood disorders, OCD) - Factors such as female gender, ethnicity, multilingualism, socio-economic factors, and more advanced language skills				

*can be administered by a non-ASD specialist, ** to be used by a specialized ASD team. M-CHAT, Modified Checklist for Autism in Toddlers; CSBS, Communication and Symbolic Behavior Scales; ADOS-2, Autism Diagnostic Observation Schedule, Second Edition; ADI-R, Autism Diagnostic Interview-Revised; AQ, Autism Quotient; EQ, Empathy Quotient; SCQ, Social and Communication Questionnaire; SRS, Social Responsiveness Scale; CAST, Childhood Asperger Syndrome Test; RAADS, Ritvo Autism Asperger Diagnostic Scale-Revised.

Regarding oncology-specific care recommendations, only very limited high-grade (i.e., prospective trial) evidence is unfortunately available on specific plans to adopt for the optimal oncological care of individuals with ASD at this time (50, 51). Nonetheless, we decided to summarize these expert-based recommendations in **Table 2**, as we feel these measures could improve care before more extensive evidence is at hand. We will discuss the main recommendations below.

**Table 2:** Recommendations for the global care planning for patients with ASD in oncology.

***Goal A: “Build a team”***
Define leading medical and nursing staff members for the patient, with whom regular face-to-face communication is performed and who act as care coordinator(s). Establish an accessible staff list of all providers involved in the patient’s care to ensure communication. Educate the caregiving team, and ancillary staff on the patient’s specific needs (by meetings and electronic chart/panels in the room) and raise awareness that the patient may be both over- and under-responsive to stimuli. Communicate regularly (scheduled meetings) with the patient and his/her family/caregiving team. Hold regular multidisciplinary team meetings with in/outpatient teams and include parents/family. Continuously reinforce collaboration among the multidisciplinary team and parents/family. Schedule satisfaction evaluations and leverage successes to build trust in caring for the patient. Allow continuity of care (same providers and environments as much as possible).
***Goal B: “Evaluate the patient’s needs & set the stage for optimal care”***
1. Prior to care, comprehensively assess personal development, daily living, skills, and sensory needs in collaboration with the family/caregivers and nursing, speech and occupational therapy teams: a. involve parents and caregiving family members in establishing inpatient and outpatient routines, b. involve and actively liaise with any outpatient care team member of the patient previously diagnosed with an ASD, c. identify preferred communication style, self-regulation abilities, specific sources of anxiety and general behavioral triggers, and potential “safe” places and objects eliciting self-regulation, d. identify common expression modalities of distress, anger, sadness and pain, e. involve a speech/language therapist for communication (e.g., develop/design pictorial boards and visual aids), f. involve an occupational therapist to assist with sensory processing difficulties, and to identify calming sensory inputs (pressure, rocking, etc.) and implement into patient room (colors, lights, objects), g. involve a child life specialist to provide emotional support, playful activities, and strategies of stress reduction 2. Prepare the patient to new environments by any aids identified above (e.g., photographic booklets of spaces to enter, social stories). 3. Eliminate potential sources of irregular, stressful, loud or unusual sounds, lights, contact experiences (incl. people). 4. Provide single rooms for patients whenever possible. 5. Discuss and establish a dietary plan featuring known and routine foods (incl. with a hospital dietician)
***Goal C: “Implement care and react to distress/crisis”***
1. Coordinate clinical care to minimize sensory input and address environmental factors that contribute to sensory processing difficulties (e.g., auditory, tactile, nutritional hypersensitivity), and busy environments (favor appointment slots in less active clinical daytime periods). Consider the possibility of consulting ASD individuals (patient experts). 2. Anticipate and group procedures, provide ample time for preparation and ensure consistency of procedures and care (maintain sequences of vital signs, clinical exams, discussions). 3. Identify/document individualized mitigation strategies that have proven to be effective. 4. Introduce cues (verbal or other) to prepare procedures or moments with potential triggers. 5. Maintain outpatient routines as much as possible while inpatient. 6. Continuously monitor for signs of imminent crisis (e.g., psychomotor agitation, increase of verbal output, stereotypies, verbal or physical aggression, self-harm). 7. In case of distress/crisis: a. Safety first: create an area of protection, immediately stop any intravenous product or non-vital procedures. b. If you feel there is the potential of losing control of the situation, immediately ask for help. c. Initiate individualized mitigation strategies. d. Perform post-hoc review of events: i. identify the triggers for the crisis (e.g., symptom, fear, discomfort, pain) ii. record the type of behavior (e.g., tantrums, screaming, self-injury, aggression) iii. document which mitigation measures and their efficacy iv. return information to healthcare team in charge of the patient

First, oncology teams need to be particularly vigilant to children, adolescents and adults with ASD, as their unique physiological and neuro-psychological (i.e., behavioral, cognitive, and motor) profiles might cause difficulties in their adherence/compliance with the “standard of care” clinical strategies in place for other patients. ASD individuals with cancer have to cognitively and emotionally grasp the stakes of a serious illness, face invasive investigations and treatments while dealing with the struggles of a neurodevelopmental disorder (52). Therefore, oncology providers need to be aware that the behavioral manifestations and the communication deficits can pose a particularly significant threat to the provision of optimal care in this very heterogeneous population (53, 54). For example, cognitive inflexibility is a key difficulty that may manifest itself in the context of cancer care, including in individuals with ASD without ID or a more subtle phenotype. In this setting, oncology teams unaware of the signs of ASD might stigmatize patients as poorly adherent/compliant or “difficult”. Overall, if not addressed, the ASD-specific features of non-standard communication and behavior carry the risk of deleteriously impacting clinical service operations and lead to unforeseen morbidity and mortality.

Second, to meet the needs of individuals with ASD and cancer, clinicians must imperatively and effectively collaborate with parent/families and other caregivers with the aims to constantly adapt communication and monitor/manage the clinical environment. It cannot be stressed enough that working in partnership with the parents/caregivers is of key importance to prioritize and coordinate interventions and treatments, and to provide adequate supportive care to the patient and his/her family members/care-proxies. This collaboration needs to be re-evaluated on a regular basis for the effectiveness in assessing patient- and caregiver-reported symptoms and issues.

Third, regarding communication, as information is being mostly provided verbally, healthcare professionals must use appropriate strategies and tools adjusted to the characteristics of the patient to facilitate transmission and understanding of information. Regardless of age and cognitive functioning level, individuals with ASD generally present strengths in the visual processing of information whereas they are often “drowned” by the flood of information presented verbally, particularly in contexts perceived as stressful. Consequently, a coherent and systematic use of visual aids by the healthcare team (e.g., images, pictures, and graphics), short visual scenarios illustrating the steps of a treatment to come, a “thermometer of emotions” to indicate the level of perceived discomfort or even pre-established diagrams/drawings at the bedside can be means to reduce anxiety and prevent a potential crisis. Examples of visual support methods related to care protocols for children and adults with ASD have been developed (55). The Autism Speaks website is a very valuable resource and provides toolkits for communication in many situations (www.autismspeaks.org/tool-kit). Knowledge on the patient’s preferred communication system (e.g., use of pictograms, images, or electronic “tablet” devices) will allow the prompt identification of needs, facilitate the integration into new or unexpected environments (incl. new providers), and allow the establishment of routines.

Fourth, establishing an individualized global care plan and specific protocols prior to the start of any exam, treatment or procedure can help the healthcare team to anticipate potential disruptions. The development of these strategies should always involve the patient, the family and/or primary caregivers and, to the extent possible, the expertise of autism specialists and individuals who themselves present ASD (47). Critical information such as ASD-specific indicators of severity, of intellectual functioning, the sensory profile, somatic and psychiatric comorbidities and whether the individual is verbal or not will need to be gathered and communicated effectively within the entire team. Oncology providers need to be aware that caregiver reports about behavioral changes or changes in physiological patterns can inform the clinical team about potential problems more swiftly than any other means. Lastly, the comprehensive and personalized care plan should be used as a tool for communication between the healthcare team members in charge of the patient, particularly at shift changes or other sign-off timepoints. **Table 2** provides an overview of our recommendations to plan, anticipate, react and report in case of behavioral crises in the clinical environment (Goal C-7). It will be important to always ensure the latter interventions can be activated swiftly in out- and inpatient settings.

Fifth, clinical environments rarely adapt to an individual sensory profile, and patients might associate oncology care with a particularly hostile experience. This is even more difficult in situations involving young children, where families play a central role as an interface. Taking the time to gradually expose the patient to the various instruments used during therapy (e.g., monitoring equipment, needles, bandages) and their sensory exploration could represent a mean to increase the chances of optimal collaboration. If, despite these arrangements, a crisis arises, the family/primary caregivers remain of crucial importance in determining the sources of discomfort. They will be able to provide insights on the warning signs of crisis and effective strategies to reduce anxiety used at home or elsewhere (e.g., stop talking, reducing noise, movement, and light, provide a sensory withdrawal room, use visual distraction, engage in the patient’s specific interests).

Sixth, oncology providers should be aware that their patients, regardless of age, might have an undiagnosed ASD and be without a definitive neurodevelopmental diagnosis at the time of the discovery of their neoplasm. In fact, even though ASD can be diagnosed as early as age 2 years, most children remain unidentified until after the age of 4 years (18). Particularly relevant for the pediatric oncologist, this is an age at which a peak in cancer diagnoses is observed. Of course, oncology teams should not be screening every patient for ASD, but consultation with in-house mental health providers or ASD specialists is indicated in cases of suspicion (see **Table 1**). As psychologists and other mental health professionals are an integral part of individuals with cancer, most oncology teams will have easy access to these resources. Nonetheless, while the first and most important reflex should always be the timely referral to specialized teams (i.e., psycho-oncology, social work, psychiatry, depending on the local setting), an improved awareness of front-line actors to the existence of standardized screening instruments (**Table 1**) could contribute to the earlier detection of ASD in situations where it does not seem apparent at first glance (56–61).

Seventh, patients with ASD often experience physical manifestations which overlap with common cancer- or cancer treatment-related symptoms, such as pain, sleep disturbances and fatigue, gastrointestinal (GI) problems or immune dysregulation (27). These symptoms can easily be exacerbated by the cancer-directed therapy or be misinterpreted. Furthermore, difficulties in communication in patients with ASD, and the lack of adapted and validated instruments, may cause difficulties in their early identification and monitoring (62). Five oncology-specific symptoms deserve special attention in individuals with ASD. They are: a) pain, b) cancer-related fatigue, c) neurological and behavioral toxicity, d) fever and e) gastro-intestinal issues. We will discuss them individually due to their high incidence/prevalence and importance for cancer-associated outcomes. Our aim for this section is mainly to provide awareness for the oncology team and hint at possible solutions to some of the challenges they can pose in individuals with ASD. More precise recommendations to manage these cancer-related symptoms in ASD specifically cannot be delineated due to the lack of evidence, and further research is urgently needed. We wish to underline that children with ASD are a) particularly at risk for misinterpretation/underestimation of these symptoms, and b) underserved by the tools available to measure them, due to many reasons whose discussion is beyond the scope of this review.

### Pain

Pain has a prevalence ranging 30–60% in oncology patients, and its screening and assessment through validated instruments is an integral part of cancer care (63, 64). However, the perception of the “experience of pain” in individuals of all ages with ASD can be difficult to assess by conventional scale measurements and research in this area is ongoing (65–67). We strongly recommend that caregivers/parents are closely involved in the assessment of pain in individuals with ASD, and when possible, self-assessment should be performed by a visual scale [e.g., adaptation of the *Face Pain Scale-Revised* (68)]. If the individual with ASD is non-verbal or has ID, hetero-rating scales will help to objectify manifestations of pain, favoring recording of scores from close parents/family members/caregivers. Although adjusted scales and measuring techniques have been developed and validated for children with cognitive limitations, data are still needed to adapt them to the specificities of ASD (69). The use of the revised *Face Legs Activity Cry Consolability* (rFLACC) pain assessment tool or the *Non-Communicating Children’s Pain Checklist Postoperative Version* (NCCPC-PV) and the inclusion of parent/family member/caregiver input in the interpretation of signs indicating pain is the minimum standard (70). Taken as a whole, the incorrect interpretation of behavioral patterns typical of individuals with ASD can lead caregivers to assign the manifestations of pain incorrectly to either an “exaggeration” or cause an underestimation of the intensity of the pain experience.

### Cancer-Related Fatigue (CRF)

Cancer-related figure (CRF) is the most prevalent symptom experienced during the cancer trajectory (incidence of 40% at diagnosis and 80–90% during chemo- and/or radiotherapy) (71). CRF is defined as a distressing, persistent, subjective sense of physical, emotional and/or cognitive tiredness or exhaustion related to cancer or cancer treatment (72). No studies or reviews published addressing how to measure and manage CRF in the ASD population, which constitutes a major issue as it significantly impacts quality of life and other treatment outcomes (71, 72). Its perception and expression are most likely altered in children and adults with ASD. In fact, associated behavioral issues can be misleading in the interpretation of CRF symptoms/signs and the communication difficulties render the differentiation of fatigue vs. other symptoms (e.g., sleepiness, dizziness, confusion, disorientation) a challenging, if not an impossible task without the collaboration of parents/caregivers. Furthermore, there is evidence to suggest that maladaptive sleep patterns in this population are associated with increased baseline daytime sleepiness, creating an additional layer of complexity (73). As it is not clear whether the current diagnostic criteria and the routine CRF management approaches appropriately address the needs of patients with ASD, we recommend that caregivers/parents are closely involved in the assessments of fatigue in patients with ASD undergoing cancer therapy. To allow the delineation of a personalized CRF-expression pattern, these evaluations need to take the individual communication/behavioral patterns into account, be compared to baseline/prior signs/symptoms and be repeated in several contexts. Once suspected, monitoring using the CRF-expression pattern should occur on a regular basis.

### Neurotoxicity

Neurological toxicity from cancer therapy has been widely recognized (i.e., headache, seizures, encephalitis, movement disorders, peripheral neuropathy) (74). With the development of immunotherapy, novel and complex patterns to identify have emerged (i.e., polyneuropathy, demyelination, leukoencephalopathy, aseptic meningitis). There is evidence of an enhanced proinflammatory profile linked with major depression, memory complaints and behavioral deficits, all of them potentially present or enhanced in the context of ASD. As neurological symptoms and cognitive deficiencies can be common side effects of cancer treatment, individuals with ASD could be at risk for underdiagnosis. Sometimes these symptoms can remain even after cessation severely impacting long-term quality of life. A similar approach to pain and fatigue should be used for neurotoxicity (see 4.1 and 4.2 above).

### Fever

Fever is a key symptom in the practice of oncology, as it can indicate infectious complications resulting from chemotherapy-induced myelosuppression. There is evidence to suggest that the manifestation of fever is altered in ASD as children with this condition rarely present with fever, and certain individuals even display an improvement of the behavioral phenotype during hyperthermia (75). The behavioral-state changes associated with fever in ASD have been speculated to depend upon the selective normalization of key components of a functionally impaired locus coeruleus-noradrenergic system (76). Therefore, oncology providers need to gather information about which signs and symptoms could indicate fever in each individual with ASD and communicate about them effectively within their teams (e.g., on-call/night shifts), as this could prevent the underreporting or underdiagnosis of potential life-threatening complications.

### Gastro-Intestinal Issues

Digestive manifestations accompanying the cancer journey include both lower (i.es., constipation, diarrhea) and upper gastro-intenstinal (GI) symptoms (i.e., nausea/vomiting, abdominal pain) with a prevalence ranging from 5–90% depending on treatment modalities (77–80). In children with ASD, diarrhea and constipation are among the most frequently reported (62, 81–85). Moreover, medications administered to patients with ASD (β-blockers and α2 agonists, dopamine-receptor blockers, opioid antagonists, anticonvulsants, etc.) can also influence gut function. Given the communication difficulties in individuals with ASD, any atypical behavior (sleep disorders, irritability, food intolerance, self-injurious behavior, posturing, grimacing, holding the abdomen, squeezing the legs together, or walking around with a narrow gait to hold the stool in) should trigger an evaluation for constipation. Furthermore, little is known about the subjective experience of chemotherapy-induced nausea/vomiting (CINV) in individuals with ASD. For oncology providers, excessive nausea/vomiting to minimally or even non-emetogenic drugs could constitute a “red flag” for ASD in a patient with other suggestive signs (see **Table 1**).

## Research on the Delivery of Cancer Care in Individuals With ASD

There is an urgent need for studies on the optimal cancer care for patients with ASD. Clinical investigation should focus on a) delineating the impact of cancer- and its therapy (incl. supportive care) on these individuals specifically, b) designing and testing adapted instruments and/or strategies to measure symptoms (e.g., CRF), c) adjusting the clinical environment to facilitate care, and d) targeting providers and healthcare systems for the delivery of effective training. Prospective trials should be conducted with the participation and close collaboration of direct caregivers (parents, families, etc.).

## Limitations and Conclusions

The main limitation in our review is the lack of study-based evidence on the cancer care of children, adolescents and adults with ASD. Thus, an extensive, systematic review of the evidence is not feasible at this time. As the number of pediatric and adult patients with ASD in our oncology practices increases, our recommendations at this time largely remain extrapolations of available data from other clinical settings.

Nonetheless, we firmly believe that this should not hinder the discussion of concrete measures that, if implemented, can immediately benefit patients with ASD undergoing cancer treatment. Giving providers (medical, nursing and affiliated staff) the opportunity of improving their care of individuals with ASD is a global priority. In the absence of higher-grade evidence, they are intended as a call for vigilance of the particularities of these highly vulnerable individuals. Further research is urgently needed in order to improve our understanding of the impact of cancer- and its therapy (incl. supportive care) on individuals with ASD, which in turn shall help to develop instruments/strategies reliably measuring quality and outcomes in this population during their cancer care trajectory.

## Author Contributions

DV, SC-L, P-AF, and RR reviewed the available evidence and took the lead in writing the manuscript. All authors contributed to the article and approved the submitted version.

## Conflict of Interest

The authors declare that the research was conducted in the absence of any commercial or financial relationships that could be construed as a potential conflict of interest.
