# Supplementary material for: Cancer Care of Children, Adolescents and Adults With Autism Spectrum Disorders: Key Information and Strategies for Oncology Teams
Source: Front Oncol. 2021 Jan 19;10:595734. doi: 10.3389/fonc.2020.595734 (PMC7856416; doi:10.3389/fonc.2020.595734)
Supplement: Supplementary file 1 [file DataSheet_1.docx]

***Supplementary Material 1***

**Areas of expertise of the authors**

1. Specific expertise in the diagnosis, care of, and research on ASD, all authors from the Division of Autism Spectrum Disorders and Related Conditions, Department of Psychiatry, Lausanne University Hospital, Switzerland.

- Delphine Vuattoux, PsyD, PhD, Clinical and Research Psychologist, Attending Staff Psychologist
- Pierre-Alain Fernandez, MD, PhD, Board-certified Psychiatrist, Attending Staff Physician
- Sabine Manificat, MD, Board-certified Pediatric Psychiatrist, Attending Staff Physician
- Marine Jequier Gygax, MD, Senior Lecturer, Board certified Pediatrician/Pediatric Neurologist, Attending Staff Physician,
- Nadia Chabane, MD, PhD, Professor, Board-certified Psychiatrist, Division Chief and Chair in Autism, Attending Staff Physician

1. Specific expertise in nursing and nursing research specifically to children/adults with cancer, all authors from Institute of Higher Education and Research in Healthcare, University of Lausanne, Switzerland

- Sara Colomer-Lahiguera, PhD, Post-Doctoral Research Scientist, with additional affiliation to the Department of Oncology, Lausanne University Hospital, Switzerland
- Anne-Sylvie Ramelet, PhD, Professor, with additional affiliation to Department “Woman-Mother-Child”, Lausanne University Hospital, Switzerland
- Manuela Eicher, PhD, Professor, with additional affiliation to the Department of Oncology, Lausanne University Hospital, Switzerland

1. Specific expertise in pediatric oncology (diagnosis, care and research), all authors from the Pediatric Hematology-Oncology Unit, Division of Pediatrics, Department “Woman-Mother-Child”, Lausanne University Hospital, Switzerland.

- Marie-Louise Choucair, MD, Board-certified Pediatrician, Junior Staff Physician
- Maja Beck-Popovic, MD, Professor, Board-certified Pediatrician/Pediatric Hematologist-Oncologist, Unit Chief & Staff Physician
- Manuel Diezi, MD, Board-certified Pediatrician/Pediatric Hematologist-Oncologist, Board-certified Pharmacologist/Toxicologist, Attending Staff Physician
- Raffaele Renella, MD, PhD, Senior Lecturer, Board-certified Pediatric Hematologist-Oncologist, Attending Staff Physician

1. Specific expertise in medical oncology (diagnosis, care and research), author from the Department of Oncology, Lausanne University Hospital, Switzerland.

- Sofiya Latifyan, MD, Board-certified in Medical Oncology, Attending Staff Physician
